# Video adjudication in clinical trials: enabling distribution of expertise and accurate scoring

**DOI:** 10.1186/1745-6215-16-S2-O60

**Published:** 2015-11-16

**Authors:** Alan Stevenson, Sharon Kean, David Cowan

**Affiliations:** 1 University of Glasgow, Glasgow, UK

## Background

Within stroke trials, there is a need to provide fair, unbiased scoring of participant assessments, based on their recovery over specified time points. In order to provide this, a key stumbling block can be assembling a reliable team of adjudicators, and coordinating that adjudication. If relying on a more distributed group, the issues then include providing access to paper notes, and tracking their comments out with a centralised meeting.

## Methods

By capturing assessments on video and developing a web based system to manage those videos, we can allow study coordinators to manage these assessments, receiving them from multiple centres and distributing them to adjudicators regardless of geographic location.

By allowing adjudicators to actually view the assessments, rather than rely on written notes, we remove a layer of obfuscation from the adjudication, with adjudicators being able to directly judge participant reactions for themselves.

We can provide a toolset embedded within a web based data capture system that can assign algorithms to the selection of adjudicators by criteria such as location, or provide translations of foreign language assessments, simplifying the work in distributing the assessments. We can also provide integration with study eCRFs, including third party providers, to report adjudicated scores back to the main study datastore, while maintaining metrics on the differences in scoring, leading to quality checks on the assessments being done in centres.

## Conclusion

We will discuss the approach of designing a generic, multi-trial system to accomplish these goals, and real life experience from its operation in multiple concurrent trials.

